# Socioeconomic inequalities in childhood overweight: heterogeneity across five countries in the WHO European Childhood Obesity Surveillance Initiative (COSI–2008)

**DOI:** 10.1038/ijo.2016.12

**Published:** 2016-03-01

**Authors:** L Lissner, T M A Wijnhoven, K Mehlig, A Sjöberg, M Kunesova, A Yngve, A Petrauskiene, V Duleva, A I Rito, J Breda

**Affiliations:** 1 Section for Epidemiology and Community Medicine (EPSO), Department of Public Health and Community Medicine, University of Gothenburg, Gothenburg, Sweden; 2 Division of Noncommunicable Diseases and Promoting Health through the Life-Course, WHO Regional Office for Europe, Copenhagen Ø, Denmark; 3 Department of Food and Nutrition and Sport Science, University of Gothenburg, Gothenburg, Sweden; 4 Obesity Management Centre, Institute of Endocrinology, Prague, Czech Republic; 5 School of Hospitality, Culinary Arts and Meal Science, Örebro University, Grythyttan, Sweden; 6 Department of Preventive Medicine, Faculty of Public Health, Lithuanian University of Health Sciences, Kaunas, Lithuania; 7 Department of Food and Nutrition, National Center of Public Health and Analyses, Sofia, Bulgaria; 8 Instituto Nacional de Saúde Dr Ricardo Jorge, Lisbon, Portugal

## Abstract

**Background:**

Excess risk of childhood overweight and obesity occurring in socioeconomically disadvantaged families has been demonstrated in numerous studies from high-income regions, including Europe. It is well known that socioeconomic characteristics such as parental education, income and occupation are etiologically relevant to childhood obesity. However, in the pan-European setting, there is reason to believe that inequalities in childhood weight status may vary among countries as a function of differing degrees of socioeconomic development and equity.

**Subjects and Methods:**

In this cross-sectional study, we have examined socioeconomic differences in childhood obesity in different parts of the European region using nationally representative data from Bulgaria, the Czech Republic, Lithuania, Portugal and Sweden that were collected in 2008 during the first round of the World Health Organization (WHO) European Childhood Obesity Surveillance Initiative.

**Results:**

Heterogeneity in the association between parental socioeconomic indicators and childhood overweight or obesity was clearly observed across the five countries studied. Positive as well as negative associations were observed between parental socioeconomic indicators and childhood overweight, with statistically significant interactions between country and parental indicators.

**Conclusions:**

These findings have public health implications for the WHO European Region and underscore the necessity to continue documenting socioeconomic inequalities in obesity in all countries through international surveillance efforts in countries with diverse geographic, social and economic environments. This is a prerequisite for universal as well as targeted preventive actions.

## Introduction

The inverse socioeconomic gradient in childhood obesity was reported as early as 1972 by Stunkard *et al.*,^1^ who observed a sixfold higher prevalence of obesity among American girls in low income than in more affluent areas and found a similar but weaker associations in boys. In subsequent years, this observation has been followed by numerous reports of the same phenomenon in other high-income countries. A review of the evidence in 2006 concluded that associations between socioeconomic status and childhood obesity have become predominantly inverse and that positive associations have all but disappeared.^2^ Other investigators have systematically reviewed the literature to investigate whether obesity interventions in children are likely to reduce rather than increase these persistent socioeconomic inequalities.^3^ The purpose of the present study is to re-examine socioeconomic differences in overweight and obesity in primary-school children from five European countries with attention to whether positive (as well as negative) associations may still exist within Europe. It was hypothesized that the nature of inequalities in childhood obesity may differ profoundly between countries with different socioeconomic features, including indices of economic inequality, gender equality and human development.

The Childhood Obesity Surveillance Initiative (COSI) was initiated by the World Health Organization (WHO) Regional Office for Europe and some Member States. The key rationale for the start of COSI was the urgent need for nationally representative and highly standardized data on childhood obesity prevalence in different European countries, as many available prevalence estimates are based on regional or convenience sampling and used varying examination protocols. According to the first report from COSI, the prevalence of childhood obesity in 12 European countries ranged from 6.0% to 26.6% in boys and from 4.6% to 17.3% in girls.^4^ Obesity progresses with increasing age, thus even higher overall obesity rates (20–25%) occur in European adults, as recently estimated by WHO.^5^ The vision of COSI is to establish a European system to routinely measure the body weight and body height of primary-school children in order to monitor progress with curbing the obesity epidemic in this population group.

## Methods

### COSI project

During the school year 2007/2008, COSI conducted its first round of anthropometric examinations in primary-school children in all participating European countries. In addition, five countries participating in this round collected supplementary data on socioeconomic characteristics of the children's families through an optional COSI family record form.^6^ The COSI protocol^7^ is in accordance with the International Ethical Guidelines for Biomedical Research Involving Human Subjects.^8^ Depending on circumstances in each country, local protocols were approved by the appropriate ethics committees: Bulgaria: the Medical Ethical Committee of the former National Center of Public Health Protection; Czech Republic: the Ethical Committee of the Institute of Endocrinology; Lithuania: Lithuanian Bioethics Committee; Portugal: Portuguese Data Protection Authority; and Sweden: regional ethics review boards in Gothenburg and Stockholm. Parents were fully informed about all study procedures; informed consent was obtained using either an active or passive approach; and confidentiality of all collected and archived data was ensured. Children's consent was always obtained prior to the anthropometric measurements.^4, 7^ Investigators in each country decided on the specific dates for taking measurements, within the defined baseline data collection period. Taking into account the local arrangements and available budgets, countries chose the most appropriate professionals to measure the children's weight and height (e.g. physical education teachers, nationally or regionally based health professionals). The family record form was either presented to parent and child prior to measurement, sent home with the child, or mailed directly to the home and was generally completed by the children's caregivers jointly with their child. A more complete description of the implementation characteristics of the first COSI round can be found elsewhere,^4, 6^ and details of the family record form have been presented on country-specific basis (for example, Moraeus *et al.*^9^).

### Sampling of children

Cluster sampling was applied using as primary sampling unit the primary school, except for the Czech Republic, where the primary sampling unit was composed of pediatric clinics, as COSI was attached to mandatory pediatric health examinations. Primary schools were selected randomly from the list of public, private and special primary schools, centrally available in each country through the ministry of education or at the national school registry or as in the Czech Republic the national list of primary care pediatricians. If all children of the specifically targeted age group were in the same grade, then one class per school was drawn within a grade level. If the specifically targeted age group was spread across grades, however, all grades where children from this age group were present could be sampled. COSI targets 6-, 7-, 8- and 9-year-old children but countries could focus on one or more of these four age groups.^7^ Detailed sampling characteristics have been described elsewhere.^4, 6^ For the purpose of the present analysis, five countries were included: Bulgaria, Czech Republic, Lithuania, Portugal, and Sweden. These five countries were the only ones fulfilling the dual criteria of having administered the optional COSI family record forms (a logistical decision of each country) in addition to measuring the children.

### Socioeconomic variables

With the family record form, data were collected on four variables reflecting parental socioeconomic position (SEP). Maternal and paternal education referred to the highest level of education that had been completed by the child's mother and father, respectively, using four answer options: primary school, secondary school, undergraduate/Bachelor degree, and Master's degree or higher.^7^ The education variable was regrouped into two categories: lower education (primary school or secondary school) and higher education (undergraduate/Bachelor degree or Master's degree or higher). Maternal and paternal occupation referred to a description of the mother's and father's 'main work over the past 12 months', respectively, using eight answer options: government-employed, non-government employed, self-employed, student, homemaker, unemployed–able to work, unemployed–unable to work, and retired. This occupation variable was regrouped into three categories: unemployed–but able to work, employed (government-employed, non-government employed or self-employed), and miscellaneous (student, homemaker, unemployed–unable to work or retired). Selected national indicators related to each country's social and economic development status in the year 2008 were also considered for descriptive and analytic purposes. These include the Gini coefficient of inequality,^10^ the Human Development Index^11^ and the Gender Inequality Index,^12^ abbreviated here as Gini, HDI and GII, respectively.

### Anthropometry

Weight and height were measured by personnel who had been trained in WHO's standardized techniques.^13^ Children removed their shoes and socks as well as heavy clothing. Body weight was measured to the nearest 0.1 kg with portable digital (generally manufacturer-calibrated) scales (SECA 872, SECA 862, SECA Bella 840 or SECA Bellissima 841 (Hamburg, Germany), Tanita UM-072 (Tokyo, Japan) or Beurer PS07 (Ulm, Germany)) and body height was measured standing upright to the nearest 0.1 cm with portable stadiometers (SECA 206, SECA 214 or SECA Leicester (Hamburg, Germany), TB I Hyssna 4205 (Strömkulla, Sweden)). Body weight was adjusted for the weight of the clothes worn, and body mass index (BMI) was calculated using the formula: adjusted weight (kg) divided by height squared (m^2^). The 2007 WHO BMI-for-age (BMI/A) distributions for schoolchildren were used to compute BMI/A *Z*-scores.^14^ Based on the BMI/A *Z*-score value, thinness was defined as <–2, normal weight between ⩾–2 and ⩽+1, overweight >+1 and obesity >+2. Throughout this paper, the group with overweight children also includes obese children, in accordance with the WHO definition of overweight.^13, 15^

### Statistical analysis

The present sample was restricted to 6.5–8.5-year-old children from five countries, with complete information on age, weight and height, with biologically plausible BMI/A *Z*-score values (between –5 and +5) ^15^ and with information on all four parental SEP variables. Descriptive statistics included country-specific mean and median BMI values, prevalence estimates for anthropometric indicators and percentages of each of the regrouped parental education and occupation categories. Lower maternal and paternal education were compared with reference groups with higher education, and maternal and paternal unemployment (excluding those unable to work) were compared with respective reference groups with employment. Logistic regression of weight status on all 4 SEP variables was performed and results were given in terms of odds ratios (OR) with 95% confidence intervals (CI) both for the unadjusted model and further adjusted for child's age, sex, and country.

Possible modifying effects of country-based indicators on the overall association between parental education or parental occupation and children's weight status were examined. Country was modelled as a categorical variable or as quantitative country-level indicator variables, that is, Gini, HDI and GII, based on published national values.^10, 11, 12^ To assess the importance of the interaction between country and parental SEP, the likelihood ratio test was used comparing the logistic model with and without the respective product term(s) describing each interaction. In the same way, interactions were tested between gender of the child and the four parental SEP variables for obesity as well as overweight within each country. Based on the results of interaction analyses, it was considered necessary for all subsequent analyses to stratify by country but not sex of the child. The final country-specific analyses were therefore adjusted for sex and age.

## Results

### Participation

In total 19 494 children were present on the day of their weight and height measurements. The children's refusal rate was the highest in Bulgaria (13%) and Sweden (12%). The complete sample of children with anthropometric examinations (*n*=18 333) was larger than the number in the final analytical sample (*n*=12 189). The first levels of attrition occurred for children whose family forms were not returned at all or if data on the parental socioeconomic variables were incomplete. Additional exclusions were made by investigators if children's ages or BMI/A *Z*-scores were outside the ranges defined in this study. As shown at the top part of Table 1, these rates can be calculated for each country. Participation analysis revealed no differences in the prevalence of overweight or obesity between groups with and without missing data, except in Lithuania where overweight was more prevalent in children with any missing information on parental SEP variables, compared with the others (not shown). Table 1 also presents anthropometric characteristics of the children in the analytical sample, while Table 2 presents parental and national-level socioeconomic characteristics for each survey country.

### SEP and overweight in girls and boys

The initial analysis was based on the full data set combining boys and girls in all countries. Table 3 shows crude and adjusted associations between the four parental SEP variables and overweight. Low education of the mother or father was associated with increased odds of overweight but the association was attenuated after adjustment for country and child's age and sex. In contrast, in both crude and adjusted models, having an unemployed but able to work parent was associated with significantly decreased risk of overweight.

The accompanying sex-stratified analysis, shown in the right columns of Table 3, suggested only minor differences between boys and girls when comparing prevalence odds ratios for overweight as a function of parental SEP. Although there was a trend for the odds ratio point estimates to be slightly stronger for girls than for boys, the associations were always consistent in both boys and girls, with confidence intervals indicating no major differences.

### Effect modification by country, country-level indicators and gender

In contrast to the gender-stratified results, visual inspection of the results after stratifying by country suggested the presence of important discrepancies between associations in the five countries. Therefore, the next step was formal testing of interactions to investigate whether the association between SEP and overweight or obesity differed among countries. For all four parental SEP variables, significant interactions with country were observed for both overweight and obesity. The results for overweight and obesity are noted as interaction *P*-vales on the far right column of Table 4. Significant interactions with respect to overweight and obesity, respectively, could also be demonstrated when the categorical country-indicator variable for each country was replaced by one of the three continuous country-level variables Gini, HDI and GII (data not shown). Use of a categorical indicator for country yielded a somewhat stronger evidence of effect modification compared with quantitative country-level indicators of socioeconomic development. In contrast to the country-indicator-based interactions, the gender interaction within each country did not reveal differences between boys and girls in the effects of parental SEP on overweight or obesity, in agreement with the initial country-aggregated results. Based on all interaction results reported so far, it was decided to combine girls and boys in the multivariable analyses while continuing to consider countries separately.

### Country-specific analyses

**Figure 1:**
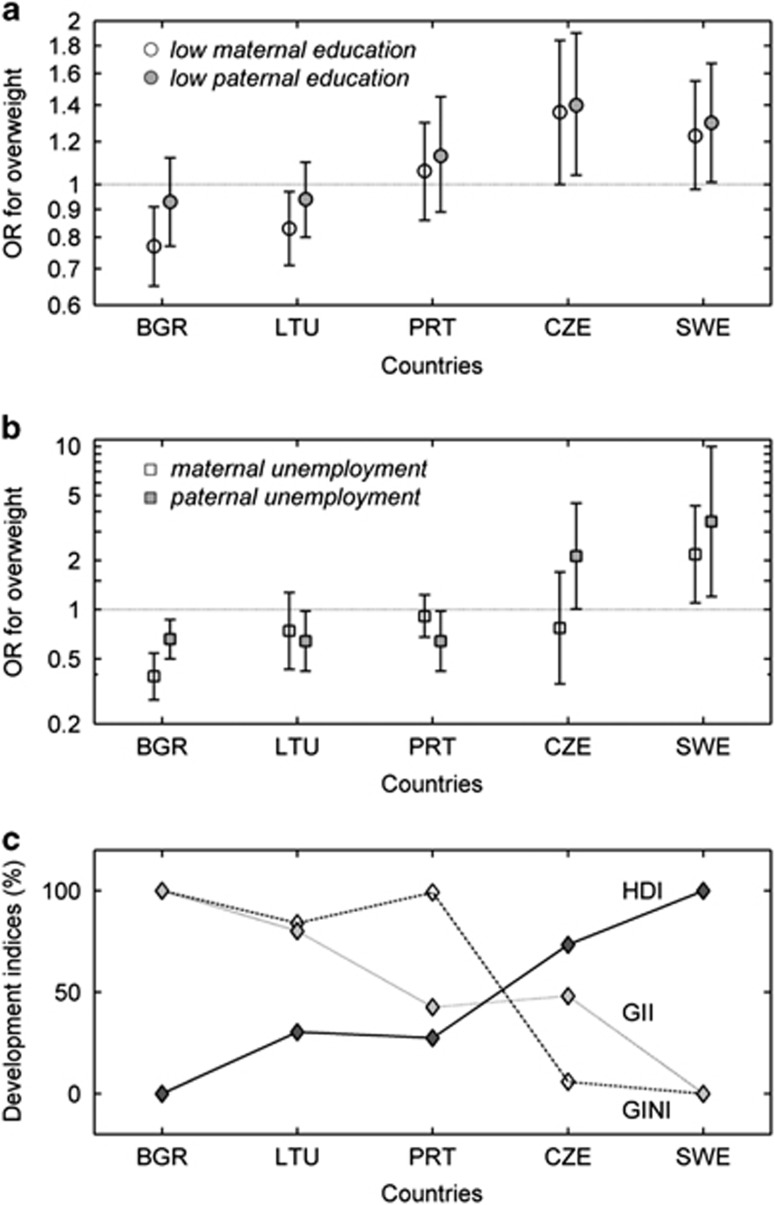
(**a**) Prevalence odds ratio for overweight (including obesity) with low maternal and paternal education, compared with reference group with higher education adjusted for sex and age. (**b**) Prevalence odds ratio for overweight (including obesity) with maternal and paternal unemployment, compared with reference group with employment; adjusted for sex and age. (**c**) Plot of country-level social indicators (HDI, GINI, GII) in each country, which have been re-scaled to vary between 0% (lowest value) and 100% (highest value), in order to ease the comparison of their variation across the five countries. Absolute values of GINI, HDI and GII at the time of the survey are presented in Table 2. Note: The country codes refer to the International Organization for Standardization (ISO) 3166-1 Alpha 3 country codes: BGR: Bulgaria, CZE: Czech Republic, LTU: Lithuania, PRT: Portugal, SWE: Sweden.

Figure 1 illustrates the heterogeneity of the socioeconomic differences for overweight, as well as the corresponding country indicators (HDI, GII and Gini) that display a similar pattern across countries to the patterns in overweight. The key point here is that the initial country-aggregated result (Table 3) masked profound heterogeneity across the five different countries, which is apparent in Figure 1. The most consistent evidence for an association between low SEP and overweight was found in Sweden, the country with the lowest inequality index, highest HDI and lowest GII. In contrast, the largest positive associations, indicating less overweight with lower SEP, was seen in Bulgaria, where country-level indicators tended towards the other extreme. The remaining three countries had mostly intermediate scores for the three country-level indicators and weaker associations with parental SEP. Finally, the figures illustrate that there was general agreement between results obtained by paternal and maternal indicators of education and employment.

In addition to country-specific results for overweight including obesity plotted in Figure 1, Table 4 also shows the prevalence odds ratios for obesity *per se* in relation to each of the four parental SEP variables. In Swedish children, low education of either parent was strongly associated with more obesity, while only in children with unemployed mothers showed higher odds for obesity. Low education of either parent was associated with more obesity in Portugal as well, whereas no statistically significant associations between parental employment and obesity were observed. In Bulgarian children, the opposite pattern was observed, with lower odds of obesity in children of less educated mothers and an even stronger association with maternal or paternal unemployment. In the Czech Republic, paternal unemployment was the only indicator that was associated with an increased risk of obesity. No statistically significant associations were observed between each of the four SEP variables and obesity in Lithuania. Comparing these results for obesity with those for overweight shown in the top portion of the table, some of the estimates differ but a similar inversion of the association across the five countries is still clearly observable. In the three countries with results indicating more obesity in children with less educated parents, even stronger associations were found for obesity.

Using country-specific odds ratios for the association between socioeconomic indicators and overweight and obesity as well as prevalence estimates for socioeconomic indicators, as given in Tables 1 and 2, it is possible to calculate attributable fractions. For instance, in Sweden, attributable fractions of obesity would be 0.48 for low maternal education but only 0.06 for maternal unemployment (data not shown), due to the low prevalence of maternal unemployment in Sweden. In less affluent countries, the fact that the odds ratio was <1 precluded the calculation of a corresponding attributable fraction. Given the heterogeneity of the associations, such calculations need to be adapted to each country-specific context.

## Discussion

In summary, the present study revealed heterogeneity in the social inequalities for childhood overweight and obesity in five countries located in different parts of the WHO European region. This modification of the association between SEP and childhood overweight/obesity was demonstrated with statistically significant interactions between parental SEP and country-based indices. The inversion of the association when moving between countries of lower and higher HDI, GII and Gini implies that inaccurate and/or attenuated estimates will be obtained when aggregating multi-country data of this type, and relevant information on socioeconomic inequalities in different regions may therefore be lost. In this study, an apparent reduced risk of overweight in children with unemployment parents in the pooled sample was confirmed individually in three countries, but the reverse was clearly observed in Sweden. The country differences in associations between socioeconomic indicators and childhood overweight were mirrored by the countries' variations in societal inequalities, using indices of inequality, human development and gender equality. In all countries, there were only minor differences in these associations when comparing boys and girls.

A main advantage of this study is the use of nationally representative sampling for recruitment of children, which can be used to generate national prevalence levels as well as to study associations between SEP and weight status. Similar associations have frequently been described based on convenience sampling, for instance, in the ‘Identification and prevention of Dietary- and lifestyle-induced health EFfects In Children and infantS (IDEFICS) study', which was started in eight European countries at approximately the same time as COSI.^16^ Although it is likely that prevalence odds ratios will give unbiased estimates of associations between SEP and obesity even in non-representative samples of schoolchildren, nationally representative prevalence estimates can potentially improve calculation of the population's attributable risk. Aside from this key difference, the COSI and IDEFICS studies had a number of strengths in common, including high standardization of instrumentation, as well as measurement of children from different parts of Europe by trained personnel.

However, the COSI study is not without limitations. For instance, in the context of representative sampling procedures mentioned above, it is acknowledged that the varying participation rates in the different countries may have resulted in selection biases when comparing prevalence odds ratios across these countries. Moreover, some of the within-country results had wide confidence limits, reflecting that sample size limitations were, in part, due to restricting the sample to families with complete information on all four socioeconomic indicators. Also owing to sample size considerations, a dichotomous parental education indicator was used, which limited any conclusions on the full educational gradient. Regarding children with low BMI/A *Z*-scores, who were included in the non-overweight group, it should be noted that there were relatively few children with any degree of thinness. It is important to emphasize that this lower side of the BMI spectrum will require further attention when extending COSI to the whole WHO European Region, particularly in countries where lower socioeconomic conditions are associated with less overweight and obesity. Finally, it is acknowledged that these results from the first COSI data collection round in 2008 were somewhat delayed and may not reflect the current situation in 2015. Nevertheless, these results represent the baseline of this WHO surveillance initiative and will therefore be the starting point for tracking future trends in socioeconomic inequalities in overweight and obesity in children.

There has been much debate about the paradoxical persistence of major health inequalities in modern welfare states, and a number of explanations have been proposed.^17, 18^ For instance, imbalances between energy intake and physical activity are believed to have a number of social and economic determinants,^18^ which could contribute to the socioeconomic inequalities in overweight and obesity. Considering the present results, the varying associations observed in the five countries indicate that inequalities in access to material and immaterial resources have not been eliminated, one of several suggested explanations by Mackenbach.^17^ From the perspective of the WHO European Region, constituting 53 Member States in various phases of economic growth, it can only be speculated how the socioeconomic gap in childhood obesity will evolve in the next decades. This situation underscores the necessity to continue documenting trends in children's obesity and related health indicators by means of international monitoring efforts.

Beyond implications for integrating surveillance in the public health commitments of European countries, these findings have interesting implications for population-based prevention of obesity. It has been suggested that well-intentioned health-promotion interventions may potentially result in disproportionate beneficial effects in the more advantaged sectors of the population.^19, 20^ If this were the case following public health initiatives to prevent obesity in the whole population, there is a risk that obesity would decrease or level out in the more affluent groups but continue to increase in the less advantaged sub-populations, with the net result that the socioeconomic gap would widen. Population-based surveillance may be a prerequisite for the identification of correct targets and high-risk communities, which serves as the basis for practical actions to reach high-risk children and families within all countries. As suggested by Stunkard *et al.*^1^ many years ago, such targeted initiatives may be necessary to prevent social inequalities from increasing.

## Conclusion

Europe remains a socioeconomically diverse region with widely varying socioeconomic differences in childhood overweight and obesity. At the time of the first COSI round, Sweden and, to a lesser extent, Portugal and the Czech Republic displayed the commonly observed excess rates of overweight and obesity in association with lower SEP. In contrast, surveys in Bulgaria and, to some extent, Lithuania yielded evidence of less overweight and obesity in less advantaged children. With the availability of repeat surveys conducted since this first examination, it will be possible to continue documenting these developments and reporting on nationally representative secular changes. In conclusion, the nutrition transition is still ongoing in parts of the European Region, which includes countries with a wide range of economic development. This survey, based on the initial round of COSI examinations, represents a starting point for studying the evolution of the childhood obesity epidemic in the twenty-first century from a socioeconomic perspective.

## Figures and Tables

**Table 1:** Body mass index (BMI) and its derived anthropometric indicators^a^ of primary-school children in five countries

*Country*	*Bulgaria*	*Czech Republic*	*Lithuania*	*Portugal*^b^	*Sweden*
Children who were present on the day of the height and weight measurements (*n*): total	3914	1695	4955	3592	5338
Children whose height and weight were measured (n): total	3392	1695	4948	3590	4708
Children who returned a family form (*n*): total	3427	1660	4436	3063	3711
Children with no missing information (*n*): total^c^	2950	1489	3408	2745	3062
Children included in the analysis (*n*): total (boys, girls)^d^	2917 (1449, 1468)	1415 (713, 702)	3376 (1716, 1660)	2694 (1340, 1354)	1787 (927, 860)
Age (years): mean (s.d.)	T: 7.7 (0.3) B: 7.7 (0.3) G: 7.7 (0.3)	T: 7.0 (0.2) B: 7.0 (0.2) G: 7.1 (0.3)	T: 7.8 (0.3) B: 7.8 (0.3) G: 7.8 (0.3)	T: 7.5 (0.6) B: 7.5 (0.6) G: 7.5 (0.6)	T: 7.9 (0.3) B: 7.9 (0.3) G: 7.9 (0.3)
BMI (kg m^−2^): mean (s.d.)	T: 16.7 (2.9) B: 16.7 (2.7) G: 16.8 (3.0)	T: 15.9 (2.2) B: 16.1 (2.2) G: 15.8 (2.1)	T: 16.5 (2.4) B: 16.6 (2.4) G: 16.3 (2.4)	T: 17.2 (2.6) B: 17.2 (2.5) G: 17.2 (2.7)	T: 16.4 (2.1) B: 16.5 (2.1) G: 16.3 (2.1)
BMI (kg m^−2^): median (Q1–Q3)	T: 16.0 (14.8–17.9) B: 16.0 (14.9–17.7) G: 16.1 (14.7–18.1)	T: 15.5 (14.5–16.8) B: 15.6 (14.7–16.9) G: 15.4 (14.3–16.7)	T: 15.9 (14.9–17.5) B: 16.1 (15.1–17.5) G: 15.8 (14.8–17.3)	T: 16.6 (15.3–18.4) B: 16.6 (15.4–18.5) G: 16.6 (15.3–18.4)	T: 16.0 (14.9–17.4) B: 16.1 (15.1–17.4) G: 15.9 (14.9–17.3)
BMI-for-age *Z*-score: mean (s.d.)	T: 0.37 (1.36) B: 0.37 (1.40) G: 0.36 (1.32)	T: 0.12 (1.21) B: 0.21 (1.30) G: 0.03 (1.11)	T: 0.29 (1.19) B: 0.40 (1.24) G: 0.18 (1.13)	T: 0.71 (1.21) B: 0.77 (1.28) G: 0.66 (1.13)	T: 0.25 (1.08) B: 0.30 (1.16) G: 0.20 (0.97)
Thinness (%)	T: 2.6 B: 2.8 G: 2.5	T: 2.9 B: 3.1 G: 2.7	T: 2.1 B: 1.6 G: 2.7	T: 0.8 B: 1.0 G: 0.7	T: 1.3 B: 1.5 G: 1.1
Normal weight (%)	T: 68.3 B: 68.1 G: 68.5	T: 76.6 B: 75.0 G: 78.2	T: 73.9 B: 71.4 G: 76.5	T: 61.3 B: 59.5 G: 63.1	T: 75.8 B: 73.1 G: 78.7
Overweight (including obesity) (%)	T: 29.0 B: 29.1 G: 29.0	T: 20.5 B: 21.9 G: 19.1	T: 24.0 B: 27.0 G: 20.9	T: 37.9 B: 39.6 G: 36.3	T: 22.9 B: 25.4 G: 20.2
Obesity (%)	T: 12.6 B: 13.0 G: 12.3	T: 7.0 B: 8.8 G: 5.1	T: 8.7 B: 10.1 G: 7.1	T: 14.5 B: 16.4 G: 12.6	T: 5.7 B: 7.1 G: 4.2

Abbreviations: B, boys; G, girls; Q1, first quartile; Q3, third quartile; T, total.

a 2007 World Health Organization recommended cutoffs for school-age children and adolescents.

b All regions except Madeira.

c Children with weight and height measurements and complete information on sex, age, maternal education, maternal occupation, paternal education and paternal occupation.

d Children were included if their age fell between the range of 6.45–8.54 years and if their BMI-for-age *Z*-score was within the normal range (⩾−5 to ⩽+5).

**Table 2:** Family- and country-level socioeconomic characteristics of the included children in five countries

*Country*	*Bulgaria*	*Czech Republic*	*Lithuania*	*Portugal*^a^	*Sweden*
*Family-level characteristics*			*Total group (boys/girls)*		
Maternal education (highest completed level) (%)					
Primary school or secondary school (low education)	66.7 (67.5/65.9)	72.4 (72.0/72.9)	46.2 (46.1/46.2)	83.1 (83.4/82.8)	57.5 (58.6/56.3)
Undergraduate, BSc., MSc. degree or higher (high education)	33.3 (32.5/34.1)	27.6 (28.1/27.1)	53.9 (53.9/53.8)	16.9 (16.6/17.2)	42.5 (41.4/43.7)
Paternal education (highest completed level) (%)					
Primary school or secondary school (low education)	78.0 (79.0/77.0)	71.9 (71.7/72.1)	56.4 (55.7/57.1)	88.3 (88.7/87.9)	68.1 (69.5/66.6)
Undergraduate, BSc., MSc. degree or higher (high education)	22.0 (21.1/23.0)	28.1 (28.3/27.9)	43.6 (44.3/43.0)	11.7 (11.3/12.1)	31.9 (30.5/33.4)
Parental education (%)					
Both low education	62.6 (63.8/61.4)	61.3 (62.0/60.7)	36.9 (35.8/37.9)	79.8 (80.1/79.6)	49.0 (50.8/47.1)
One high education	19.4 (18.8/20.0)	21.6 (19.6/23.7)	28.8 (30.1/27.5)	11.7 (11.9/11.5)	27.5 (26.4/28.7)
Both high education	18.0 (17.4/18.5)	17.0 (18.4/15.7)	34.3 (34.0/34.6)	8.5 (8.1/8.9)	23.5 (22.8/24.2)
Maternal occupation (main work over last 12 months) (%)					
Unemployed, but able to work	10.8 (10.5/11.1)	3.3 (2.8/3.9)	2.5 (2.8/2.2)	7.6 (8.0/7.2)	2.0 (2.2/1.9)
Employed	68.6 (68.5/68.8)	69.5 (71.1/68.0)	76.1 (76.0/76.1)	72.7 (73.0/72.4)	85.3 (86.5/84.0)
Miscellaneous^b^	20.6 (21.0/20.1)	27.1 (26.1/28.2)	21.4 (21.2/21.6)	19.8 (19.0/20.5)	12.7 (11.3/14.2)
Paternal occupation (main work over last 12 months) (%)					
Unemployed, but able to work	11.3 (11.0/11.6)	2.3 (1.7/2.9)	4.6 (4.8/4.5)	4.2 (4.5/3.8)	0.8 (0.8/0.8)
Employed	86.7 (87.4/86.0)	96.2 (97.1/95.3)	92.3 (92.1/92.5)	93.7 (93.3/94.1)	96.2 (97.2/95.1)
Miscellaneous^b^	2.0 (1.6/2.5)	1.6 (1.3/1.9)	3.1 (3.2/3.0)	2.2 (2.2/2.1)	3.0 (2.1/4.1)
Parental occupation (main work over the past 12 months) (%)					
Both unemployed^c^	9.1 (8.6/9.5)	2.0 (1.4/2.6)	1.9 (1.6/2.2)	1.9 (2.0/1.7)	0.6 (0.5/0.6)
Both miscellaneous^b^	0.9 (0.8/1.1)	0.5 (0.3/0.7)	1.4 (1.5/1.3)	1.1 (1.3/0.8)	1.5 (1.3/1.6)
One employed^d^	24.7 (25.3/24.1)	29.3 (28.5/30.2)	25.1 (25.9/24.2)	27.8 (27.0/28.5)	14.5 (12.6/16.5)
Both employed	65.3 (65.3/65.3)	68.2 (69.9/66.5)	71.7 (71.1/72.2)	69.3 (69.6/69.0)	83.5 (85.5/81.3)
					
*Country-level characteristics*			*Total group*		
Human Development Index (HDI) 2008	0.765	0.864	0.806	0.802	0.900
Gini coefficient 2008	35.9	24.7	34.0	35.8	24.0
Gender Inequality Index (GII) 2008	0.242	0.148	0.206	0.138	0.061

a All regions except Madeira.

b The 'miscellaneous' category included homemakers, students, retirees and unemployed persons who were not able to work.

c This occupation category referred to 'both unemployed–able to work' or '1 unemployed–able to work, 1 miscellaneous'.

d This occupation category referred to '1 employed, 1 unemployed–able to work' or '1 employed, 1 miscellaneous'.

**Table 3:** Association between socioeconomic position and risk of overweight (including obesity)^a^ among children aged 6.5–8.5 years (crude and adjusted OR, 95% CI) in five European countries

*Socioeconomic position*	*Total group*		*Boys*		*Girls*	
	*Crude OR (95% CI)*	*Adjusted*^b^ *OR (95% CI)*	*Crude OR (95% CI)*	*Adjusted*^c^ *OR (95% CI)*	*Crude OR (95% CI)*	*Adjusted*^c^ *OR (95% CI)*
*Maternal education*^d^						
High education	1.0 (Reference)		1.0 (Reference)		1.0 (Reference)	
Low education	1.08 (1.00–1.18)	0.94 (0.86–1.03)	1.04 (0.93–1.17)	0.93 (0.82–1.05)	1.13 (1.00–1.27)	0.96 (0.85–1.09)
						
*Paternal education*^d^						
High education	1.0 (Reference)		1.0 (Reference)		1.0 (Reference)	
Low education	1.22*** (1.11–1.33)	1.05 (0.96–1.16)	1.21** (1.07–1.37)	1.08 (0.94–1.23)	1.23** (1.07–1.40)	1.03 (0.90–1.19)
						
*Maternal occupation*^e^						
Employed	1.0 (Reference)		1.0 (Reference)		1.0 (Reference)	
Unemployed–able to work	0.78** (0.65–0.93)	0.67*** (0.56–0.81)	0.80 (0.63–1.03)	0.71** (0.55–0.92)	0.76* (0.58–0.99)	0.63*** (0.48–0.82)
						
*Paternal occupation*^e^						
Employed	1.0 (Reference)		1.0 (Reference)		1.0 (Reference)	
Unemployed–able to work	0.78** (0.65–0.94)	0.72*** (0.60–0.88)	0.91 (0.70–1.17)	0.87 (0.67–1.12)	0.66** (0.49–0.87)	0.59*** (0.44–0.78)

Abbreviations: CI, confidence interval; OR, odds ratio. *P<0.05; **P⩽0.01; ***P⩽0.001.

a Overweight children were compared against all other children.

b Adjusted for sex, age and country.

c Adjusted for age and country.

d High education: Bachelor degree or higher: low education: primary or secondary school.

e Homemakers, students, retirees and unemployed persons who were not able to work were excluded from these analyses.

**Table 4:** Prevalence odds ratio for overweight including obesity (upper panel) and for obesity (lower panel) for the four socioeconomic position (SEP) variables, stratified by country (adjusted for age and sex) and *P*-values for tests of country–SEP interactions in country-pooled data

	*Bulgaria*	*Czech Republic*	*Lithuania*	*Portugal*	*Sweden*	p_*int*_^a^
*Overweight*^b^						
Maternal education^c^						
Low vs high (ref)	0.77** (0.65–0.91)	1.34 (0.99–1.82)	0.83* (0.71–0.97)	1.05 (0.86–1.30)	1.21 (0.97–1.52)	0.0007
Paternal education^c^						
Low vs high (ref)	0.93 (0.76–1.12)	1.39* (1.03–1.88)	0.94 (0.80–1.10)	1.14 (0.89–1.46)	1.29* (1.01–1.65)	0.042
Maternal occupation^d^						
Unemployed-able to work vs employed (ref)	0.40*** (0.29–0.54)	0.81 (0.37–1.77)	0.74 (0.43–1.27)	0.91 (0.68–1.23)	2.20* (1.11–4.35)	<0.0001
Paternal occupation^d^						
Unemployed-able to work vs employed (ref)	0.66** (0.50–0.87)	2.10 (1.00–4.41)	0.64* (0.42–0.97)	0.64* (0.42–0.97)	3.45* (1.20–10.0)	0.002
						
*Obesity*^e^						
Maternal education^c^						
Low vs high (ref)	0.79* (0.63–1.00)	1.29 (0.80–2.09)	0.97 (0.76–1.24)	1.57** (1.14–2.17)	2.62*** (1.63–4.22)	<0.0001
Paternal education^c^						
Low vs high (ref)	0.91 (0.70–1.19)	1.59 (0.96–2.64)	1.01 (0.79–1.29)	1.63* (1.11–2.40)	2.41*** (1.41–4.09)	0.003
Maternal occupation^d^						
Unemployed-able to work vs employed (ref)	0.37*** (0.23–0.61)	0.60 (0.14–2.55)	0.36 (0.11–1.13)	1.03 (0.69–1.55)	3.85** (1.54–9.61)	0.0001
Paternal occupation^d^						
Unemployed-able to work vs employed (ref)	0.53** (0.35–0.81)	3.40** (1.35–8.55)	0.55 (0.27–1.13)	0.70 (0.38–1.29)	1.30 (0.17–10.17)	0.010

a *P*-value for effect modification of SEP on overweight and obesity by country.

b Overweight including obese children were compared against all other children.

c High education: Bachelor degree or higher; low education: primary or secondary school.

d Homemakers, students, retirees and unemployed persons who were not able to work were excluded from these analyses.

e Obese children were compared against all other children.

*P<0.05; **P<0.01; ***P⩽0.001.

## References

[bib1] Stunkard A, d'Aquili E, Fox S, Filion RDL. Influence of social class on obesity and thinness in children. JAMA 1972; 22: 579–584. 5068079

[bib2] Shrewsbury V, Wardle J. Socioeconomic status and adiposity in childhood: a systematic review of cross-sectional studies 1990–2005. Obesity 2008; 16: 275–284. 18239633 10.1038/oby.2007.35

[bib3] Bambra CL, Hiller FC, Cairns JM, Kasim A, Moore HJ, Summerbell CD. How effective are interventions at reducing socioeconomic inequalities in obesity among children and adults? Two systematic reviews. Part 1: how effective are public health interventions at reducing socioeconomic inequalities in obesity among children? Public Health Res 2015; 3: 5–38.

[bib4] Wijnhoven TMA, van Raaij JMA, Spinelli A, Rito AI, Hovengen R, Kunesova M et al. WHO European Childhood Obesity Surveillance Initiative 2008: weight, height and body mass index in 6–9-year-old children. Pediatr Obes 2013; 8: 79–97. 23001989 10.1111/j.2047-6310.2012.00090.x

[bib5] World Health OrganizationGlobal status report on noncommunicable diseases 2014. World Health Organization: Geneva, Switzerland, 2014. Available from: http://www.who.int/global-coordination-mechanism/publications/global-status-report-ncds-2014-eng.pdf?ua=1 (accessed August 2015).

[bib6] Wijnhoven T, van Raaij J, Breda J. WHO European Childhood Obesity Surveillance Initiative: implementation of round 1 (2007/2008) and round 2 (2009/2010). WHO Regional Office for Europe: Copenhagen, Denmark, 2014. Available from: http://www.euro.who.int/__data/assets/pdf_file/0004/258781/COSI-report-round-1-and-2_final-for-web.pdf?ua=1 (accessed April 2015).

[bib7] Wijnhoven T, Branca F. WHO European Childhood Obesity Surveillance Initiative. Protocol, Version January 2008. WHO Regional Office for Europe: Copenhagen, Denmark, 2008.

[bib8] Council for International Organizations of Medical Sciences, World Health OrganizationInternational ethical guidelines for biomedical research involving human subjects. Council for International Organizations of Medical Sciences: Geneva, Switzerland, 2002. Available from: http://www.cioms.ch/publications/guidelines/guidelines_nov_2002_blurb.htm (accessed April 2015).

[bib9] Moraeus L, Lissner L, Yngve A, Poortvliet E, Al-Ansari U, Sjöberg A. Multi-level influences on childhood obesity in Sweden – societal factors, parental determinants, and child's lifestyle. Int J Obes (Lond) 2012; 36: 969–976. 22614053 10.1038/ijo.2012.79

[bib10] The World Bank. Inequality: methods and tools. Available from: http://web.worldbank.org/ (accessed April 2015).

[bib11] United Nations Development Programme. Human Development Index (HDI). Available from: http://hdr.undp.org/en/content/human-development-index-hdi-table (accessed April 2015).

[bib12] United Nations Development Programme. Gender Inequality Index (GII). Available from: http://hdr.undp.org/en/content/gender-inequality-index (accessed April 2015).

[bib13] World Health OrganizationPhysical status: the use and interpretation of anthropometry. Report of a WHO Expert Committee. WHO Technical Report Series, No. 854. World Health Organization: Geneva, Switzerland, 1995. Available from: http://apps.who.int/iris/bitstream/10665/37003/1/WHO_TRS_854.pdf (accessed April 2015). 8594834

[bib14] de Onis M, Onyango AW, Borghi E, Siyam A, Nishida C, Siekmann J. Development of a WHO growth reference for school-aged children and adolescents. Bull World Health Organ 2007; 85: 660–667. Available from: http://www.who.int/growthref/growthref_who_bull.pdf (accessed April 2015). 18026621 10.2471/BLT.07.043497 PMC2636412

[bib15] Blössner M, Siyam A, Borghi E, Onyango A, de Onis M. WHO AnthroPlus for Personal Computers Manual: Software for Assessing Growth of the World's Children and Adolescents. World Health Organization: Geneva, Switzerland, 2009. Available from: http://www.who.int/growthref/tools/who_anthroplus_manual.pdf (accessed April 2015).

[bib16] Ahrens W, Pigeot I, Pohlabeln H, DeHenauw S, Lissner L, Molnar D et al. Prevalence of overweight and obesity in European children below the age of 10. Int J Obes 2014; 38: S99–S107. 10.1038/ijo.2014.140 25376223

[bib17] Mackenbach JP. The persistence of health inequalities in modern welfare states: the explanation of a paradox. Soc Sci Med 2012; 75: 761–769. 22475407 10.1016/j.socscimed.2012.02.031

[bib18] Magnusson M, Sørensen TI, Olafsdottir S, Lehtinen-Jacks S, Lingaas Holmen T, Heitmann BL et al. Social inequalities in obesity persist in the Nordic region despite its relative affluence and equity. Curr Obes Rep 2014; 3: 1–15, eCollection 2014. 24533235 10.1007/s13679-013-0087-2 PMC3920028

[bib19] White M, Adams J, Heywood P. How and why do interventions that increase health overall widen inequalities within populations?In: Babones SJ (ed). Social Inequality and Public Health. Policy Press: Bristol, UK, 2009, pp 65–82.

[bib20] World Health Organization Population-Based Prevention Strategies for Childhood Obesity: Report of a WHO Forum and Technical Meeting, 15–17 December 2009. World Health Organization: Geneva, Switzerland, 2010. Available from: http://www.who.int/dietphysicalactivity/childhood/child-obesity-eng.pdf (accessed April 2015).

